# Central Oxytocin and Food Intake: Focus on Macronutrient-Driven Reward

**DOI:** 10.3389/fendo.2015.00065

**Published:** 2015-04-28

**Authors:** Anica Klockars, Allen Stuart Levine, Pawel Karol Olszewski

**Affiliations:** ^1^Department of Biological Sciences, University of Waikato, Hamilton, New Zealand; ^2^Department of Food Science and Nutrition, University of Minnesota, St. Paul, MN, USA

**Keywords:** appetite regulation, reward system, satiety, sucrose, overeating

## Abstract

Centrally acting oxytocin (OT) is known to terminate food consumption in response to excessive stomach distension, increase in salt loading, and presence of toxins. Hypothalamic-hindbrain OT pathways facilitate these aspects of OT-induced hypophagia. However, recent discoveries have implicated OT in modifications of feeding via reward circuits: OT has been found to differentially affect consumption of individual macronutrients in choice and no-choice paradigms. In this mini-review, we focus on presenting and interpreting evidence that defines OT as a key component of mechanisms that reduce eating for pleasure and shape macronutrient preferences. We also provide remarks on challenges in integrating the knowledge on physiological and pathophysiological states in which both OT activity and macronutrient preferences are affected.

## Introduction

Macronutrient composition of ingested food affects functioning of the organism during various physiological and pathophysiological challenges, such as pregnancy, lactation, and aging. A dynamic endocrine balance facilitates the coupling of mechanisms that link appetite regulation, metabolism, and cellular/tissue-specific responses, and one of the key hormonal regulators is oxytocin (OT) (1, 2). OT affects peripheral tissues directly by binding its G protein-coupled receptor localized in, to name a few, the mammary gland, ovary, uterus and bone. OT’s action at peripheral OT receptors, interplay between OT and other hormones, as well as functional relationships with metabolic regulators, have been thoroughly studied in relation to mechanisms essential for health (1, 3). However, OT affects organism’s functioning also by regulating intake of specific macronutrients. Those mechanisms are predominantly mediated via the OT receptor localized in the brain, and our knowledge of them has expanded rapidly in the past several years. We dedicate this mini review to synthesizing currently available information on the role of central OT circuits in eating behavior, with particular emphasis on shaping food preferences. Our final remarks pertain to delineating perspectives and challenges of linking seemingly unrelated outcomes of OT’s action in the brain and in the periphery.

## Central OT and Food Intake

### Early discoveries: OT decreases consummatory behavior

From the very early stages of experimental work, it became apparent that OT promotes termination of feeding associated with generalized satiation as well as stemming from consumption-related adverse phenomena that jeopardize homeostasis (4–7).

Neuroanatomical studies have shown that most OT neurons are localized in the hypothalamus, and its paraventricular nucleus (PVN) is the main source of OT fibers innervating central targets, most prominently, the dorsal vagal complex in the brain stem (8–12). Aside from the parvocellular PVN neurons, OT is also released centrally via somatodendritic projections of magnocellular OT subpopulations in the supraoptic nucleus (SON) and PVN (13, 14). Lesioning of the PVN and disruption of the PVN-hindbrain pathways lead to increased food intake and body weight in rats (15–17). Release of OT and increased activity of OT neurons coincide with satiation-associated termination of feeding in laboratory animals (6, 18–20). Arletti et al. were first to report that intracerebroventricular (ICV) injection of OT causes a marked reduction in deprivation-induced food intake in rats (18). Many authors have confirmed the finding and, by using intraparenchymal OT receptor ligand injections or employing OT receptor-specific cytotoxins, they identified the hindbrain (particularly the dorsal vagal complex) as the area through which OT-driven feeding inhibitory mechanisms are executed (21).

A number of satiety inducing neuropeptides have been shown to affect appetite, at least partially, by acting via OT containing pathways. Those peptides include – among others – alpha melanocyte-stimulating hormone (alpha-MSH) and glucagon-like peptide-1 (GLP-1), key components of the brainstem-hypothalamic appetite circuit (22, 23). Furthermore, hyperphagia and obesity occur in mutations that lead to insufficiencies in OT PVN neuronal population development, such as that observed in the single-minded-1 (sim-1) mouse model, and these negative symptoms can be reversed by OT treatment (24). A reduction in the number of OT neurons has been reported for Prader–Willi syndrome patients exhibiting extreme overeating (25, 26). Recently, the ventromedial hypothalamic (VMH) nucleus has been identified as a hypothalamic site through which OT causes early meal termination in free feeding and fasted rats (27).

It should be emphasized that the intake of a sufficient amount of energy does not appear to be the main or the necessary factor that induces OT neuronal activity underlying termination of ingestive behavior. In fact, OT neuronal activity and release coinciding with termination of feeding occur upon changes in calorie-independent parameters associated with consumption. Those parameters include excessive stomach distension and elevated plasma osmolality (28–30). In addition, central OT inhibits consumption of toxin-tainted foods and supports long-term avoidance of those by acting through not only the brain stem but also the amygdala (31).

Though the protection of internal milieu during consumption appears to be the key neuroregulatory function of OT within the CNS, its importance for facilitating important competing behaviors, particularly with regard to reproductive and social behaviors, should not be disregarded. Sexual intercourse, lactation, and bonding within family and non-family groups are well-known stimuli to cause OT secretion in various species (32–38). Interruption of these processes by the drive to consume food, under certain conditions, might not be evolutionarily advantageous. Therefore, the anorexigenic function of OT should be seen from a broader point of adjusting/balancing physiological and behavioral responses to both internal and external challenges.

### Oxytocin and reward: Protecting against overeating carbohydrates?

While the involvement of OT in the “homeostatic” regulation of food intake has been a widely recognized phenomenon, the past several years have brought exciting discoveries that strongly suggest an implication of central OT in another aspect of consumption: macronutrient preferences and feeding reward. These discoveries have capitalized on linking evidence pertaining to neuroanatomy and functional significance of the OT system outside the realm of classical satiety/feeding termination mechanisms, and they have refined our understanding of OT as not just a “homeostasis rescue molecule,” but also as a neuroregulator of intricate and complex dietary choice processes. Numerous reports have shown widespread distribution of the OT receptor throughout the brain and, importantly, specific sites involved in reward processing, such as the nucleus accumbens and the ventral tegmental area, appear to be prominent central targets of OT signaling (39, 40). PVN OT neuronal projections form somatic and axodendritic synapses with mesolimbic neurons (41, 42). Data from human and laboratory animal studies link OT receptor activation/availability with modifications in non-feeding rewards (from natural rewards, such as social and reproductive behaviors to administration of drugs of abuse). For example, Jarrett and colleagues found that cocaine treatment changes OT receptor binding density in the bed nucleus of the stria terminalis in female rats (43). Baracz et al. reported that direct intraparenchymal administration of OT in the core of the nucleus accumbens dose-dependently decreases methamphetamine-seeking behavior (44). The same group of investigators found also that intra-accumbens core OT attenuates methamphetamine-induced conditioned place preference in rats (45). In a recently published set of experiments employing OT receptor ligand injections in the nucleus accumbens and lentiviral-mediated overexpression of the OT receptor in this site, Bahi showed that OT attenuates the development, maintenance, and primed reinstatement of ethanol-induced conditioned place preference (46). Intracranial infusions of OT in female mice promote the development of a conditioned social preference (47). Damiano et al. showed by using functional magnetic resonance imaging (fMRI) that certain single nucleotide polymorphisms in the OT receptor gene are associated with a differential response of the mesolimbic system during anticipation of monetary rewards in healthy human subjects (48). Neurochemical studies have pointed to a relationship between OT and dopamine in modification of perceived rewards; for example, in mice central administration of OT has been found to reduce methamphetamine-elicited dopamine release in the striatum and nucleus accumbens (39) and promote a concomitant decrease in glutamate release and increase in extracellular presence of γ-aminobutyric acid (GABA) in the medial prefrontal cortex (49).

Neuroendocrine and behavioral processes governing food intake and addiction show a partial overlap. For example, an appetite stimulating hormone, ghrelin (50), activates the VTA dopamine circuit and promotes consumption of palatable food over “bland” diets (51), increases ethanol intake (52) and facilitates cocaine-induced conditioned place preference in rodents (53). Injections of anorexogenic leptin decrease self-administration of drugs of abuse (54), whereas food restriction has an opposite effect (55). Finally, sugar preference is associated with increased ethanol responsiveness (56), self-administration of cocaine (57), and amphetamine (58) in rats. Therefore, considering the link between central OT and addictive-like behaviors, an intuitive question arose as to whether this relationship might expand onto feeding reward.

Pioneering studies were performed on OT knockout (KO) mice. Amico and colleagues found that genetic deletion of OT leads to the enhanced initial and sustained intake of palatable sucrose solutions in the KO mice compared to the wild-type (WT) counterparts (59). The effect of the OT-null genotype on sucrose consumption could be observed in both dark and light phase of the 24-h cycle, and it persisted even in animals subjected to periods of stress induced in the platform shaker stress model (60). OT KO mice and their background strain tested in a progressive ratio operant licking paradigm display a similar motivational drive to consume sucrose (61). Sclafani et al. found that OT KOs given a choice between two tastants (water served as a control ingestant), exhibit a heightened preference not just for sucrose, but for palatable isocaloric carbohydrate solutions regardless of their sweetness (e.g., Polycose and cornstarch). Interestingly, a non-caloric non-carbohydrate sweetener, saccharin, was also overconsumed by the KOs (60), which is in line with the notion that OT affects feeding reward. Interestingly, the propensity to overconsume palatable tastants in OT KO mice does not generalize to fat. Two-bottle preference tests in which mice could choose between water and a palatable lipid emulsion, Intralipid, showed a similar fat preference profile between KO and WT cohorts (61). In order to further examine the issue of preference to fat, Miedlar et al. (62) employed a similar paradigm as the one used by Amico et al. in the initial study on sucrose intake in OT Kos; however, instead of the sugar water, the animals were given Intralipid. While OT KO mice drank more Intralipid during the first day of having access to the tastant (which may be related to altered neophobic or stress-related processing), on subsequent days they were found to consume the same amount of Intralipid as WT controls.

The OT KO model findings are largely in agreement with the results of experiments on laboratory animals without genetic modifications in the OT system. Gene expression analysis with real-time PCR showed upregulation of OT mRNA levels in the hypothalami of rats eating scheduled, volume-unrestricted, high-sugar diet compared to standard food (63). An increase in OT transcript content has been also found in mice given 48-h *ad libitum* access to a 10% sucrose solution versus animals consuming isocaloric Intralipid during that time (64). Herisson et al. studied hypothalamic OT gene expression in mice given short-term access to sucrose, cornstarch, or saccharin (on top of the standard diet) and determined that exposure to carbohydrates but not to saccharin elevated OT mRNA above control values; notably, a higher level of significance was detected after sucrose intake (65). Furthermore, the comparison of hypothalamic OT neuronal activity levels induced by consumption of sucrose or Intralipid (equivalent volumes) shows a much greater number of Fos-positive OT cells in the sucrose group. It should be noted, however, that even in the case of mice ingesting fat, OT neuronal activity is higher at the end than at the beginning of a meal, which reflects the role of central OT as a general satiety mediator and the phenomenon of elevated OT neuronal activation and OT release coinciding with feeding termination is seen regardless of a diet type and palatability (66). Diet composition does, however, affect the magnitude of the OT system’s response at the end of a meal (66–68).

Injection studies utilizing a blood–brain barrier (BBB) penetrant OT receptor antagonist, L-368,899, in both choice and no-choice feeding paradigms (68), have consistently produced elevation of carbohydrate intake in laboratory animals, whereas consumption of Intralipid has not been affected (64, 65). When a choice between carbohydrates is given in a two-bottle test, OT receptor blockade by systemic administration of L-368,899 appears to have a preferential stimulatory effect on sucrose consumption, which can either reflect a special functional relationship between central OT and appetite for this particular carbohydrate, or – in the light of recent studies showing the presence of the OT receptor in taste buds – can at least partially (via peripheral interactions of the systemically injected agent) stem from altered taste perception (64, 65, 69).

Mullis and colleagues have recently reported an important piece of evidence linking OT to feeding reward (70). They equipped rats with a cannula aimed at the ventral tegmental area and found that OT infusion in this site decreases deprivation-induced chow intake as well as palatability-driven sucrose consumption. These effects are abolished by a pretreatment with an OT receptor antagonist, L-368,899. Importantly, when L-368,899 or another OT receptor antagonist, (d(CH2)5(1),Tyr(Me)(2),Orn(8))-OT, were injected alone in the ventral tegmental area, they stimulated sugar intake, but they failed to induce chow consumption (70). This is consistent with the earlier findings showing that when animals are given a choice between sucrose and fat diets, systemic administration of a BBB-penetrant OT receptor blocker shifts preference toward sugar without affecting total energy consumption (64). Human research on the effects of OT on eating for reward is still very much in its infancy. It is known that in pregnant women, food cravings diminish as OT levels increase gradually during gestation (71). Over the course of the menstrual cycle, food intake (including sugar) is low during ovulation (high OT levels) and it increases during the luteal phase (low OT levels) (72, 73). The 2013 paper by Ott and colleagues (74) outlined the effects of intranasally administered OT on ingestive behavior, with special emphasis on rewarding aspects of consumption. OT was found to markedly decrease snack consumption (chocolate cookies, rice waffles, and salt crackers were offered to the subjects) during the postprandial snack test administered shortly after a full buffet-style breakfast. Noteworthy, the reduction in total snack intake was driven primarily by restraining by 25% the consumption of high-sugar chocolate cookies; hence, the initial human data support the notion coined through laboratory animal experiments that there is a functional link between OT and sugary food-driven reward. Obviously, one of the key issues that warrants caution in how we interpret the currently available body of evidence (especially related to human observations) is that while both dendtritic and hypophyseal relaease of OT can occur simultaneously, peripheral levels of OT do not always correlate with central secretion (75–78). Furthermore, McCullough and colleagues have recently provided a comprehensive analysis of pitfalls associated with typically used research techniques and strategies aimed at determining concentration of peripheral OT, and urged a particularly careful approach to construing peripheral OT data (79).

Intriguingly, while central OT suppresses certain types of feeding reward (especially those related to carbohydrate consumption) and contributes to a reduction of intake and a transient shift in dietary choices, it appears that orexigenic opioid receptor ligands (and possibly also other neuromediators of feeding for pleasure) diminish meal-end activity of hypothalamic OT neurons thereby likely promoting continuation of ingestive behavior. For example, butorphanol tartrate at a dose that promotes overeating of sugary foods dampens OT PVN neuronal activity in rats that have consumed the amount of high-sucrose powder diet that is satiating for saline-treated controls (80). In rats, opioid receptor agonists have also been shown to decrease OT neuronal activity in response to noxious stimuli, whereas an antagonist, naloxone, potentiates anorexigenic effects of emetic agents (81, 82). Finally, Mitra et al. showed that daily habitual intake of high-sucrose diets in rats reduces c-Fos expression in OT neurons after a high-sucrose or low-sucrose meal compared to rats receiving daily low-sugar food (83). This suggests that regular sugar consumption might reduce the meal-end activity of OT neurons in response to any food regardless of its composition. Hence, the balance of evidence suggests that while endogenous OT appears to reduce the consumption of palatable sugary foods, habitual ingestion of such foods – typically associated with enhanced activity in reward circuits – may dampen responsiveness of OT neurons to physiological parameters that would otherwise be conducive to termination of food intake.

## Perspectives: Central OT, Food Intake, and Aging

**Figure 1:**
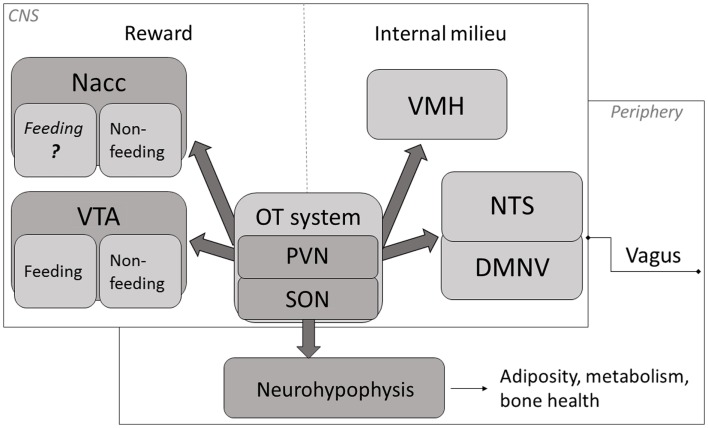
**A schematic representation of key mechanisms through which OT affects appetite**. OT neuronal activity has been associated with termination of food intake (84–87). The magnitude of this response is modified by the integrated peripheral signals (mediated largely via the vagus and the brainstem relay circuit) and by the rewarding value of a meal (74, 84, 88, 89). Release of OT in the CNS promotes termination of consumption when internal milieu is jeopardized (energy imbalance, abnormal GI and/or osmotic parameters) as well as part of intricate reward processing. Neurohypophyseal OT participates in the regulation of mechanisms related to metabolism, adiposity and bone tissue status. Nacc, nucleus accumbens; VTA, ventral tegmental area; PVN, paraventricular nucleus; SON, supraoptic nucleus; VMH, ventromedial hypothalamus; NTS, nucleus of the solitary tract; DMNV, dorsal motor nucleus of the vagus.

Evidence indicates that, aside from facilitating generalized feeding termination, central OT under certain circumstances plays a role of a carbohydrate (especially, sugar)-specific satiety mediator and, therefore, it may influence the composition characteristics of a freely selected daily diet (a schematic overview of key anatomical components of OT-dependent feeding mechanism are depicted in Figure 1). Thus, changes in OT system’s activity that modify selection of foods due to their macronutrient content may affect health status of the organism.

Aging is associated with disturbances in food intake, notably with a decrease in energy consumption and anhedonia (90), and those have been attributed to psychosocial and pathophysiological causes. Data generated through human observations and laboratory animal models reflect the age-related decline in food intake and the dysregulation of energy balance (91–94). Importantly, it has been shown that in human beings (95) and in rodents (96), fat preference is greatly diminished, whereas the percentage intake of carbohydrate-derived calories is elevated (97, 98). While the shift in macronutrient preferences likely reflects changed metabolic needs, it may simultaneously contribute to susceptibility to the development of age-related pathologies: potential consequences of alterations in macronutrient intake are extremely broad and may be conducive to energy imbalance (99), fat mass changes (100–103), and osteoporosis (104–106).

One of the greatest challenges is to identify in which of the many physiological and pathophysiological states associated both with shifts in dietary preferences and changes in OT system’s activity, the modified OT tone serves as the causative factor of undesirable modifications in a consumption profile. Unfortunately, our knowledge of changes in the OT system in aging, especially those within the central nervous system, is far from being systematized. The relatively few studies published thus far have presented conflicting evidence in regard to the density of the OT receptor, number of OT neurons, and functional outcomes of exogenous and endogenous OT during the aging process. For example, Fliers and Swaab (107) reported an age-related increase in OT secretion in the PVN, but not in the SON, whereas OT plasma levels were similar in young versus old male rats (107). Keck et al (90) found a decrease in stress-induced intra-PVN OT secretion in male rats, while Zbuzek et al (108) and Melis et al (109) did not detect differences in hypothalamic OT levels (90, 108, 109). An age-related decrease in OT concentration was shown in the septum and hippocampus, and a decrease in OT receptor binding, in the caudate putamen, olfactory tubercle, and ventromedial hypothalamic nucleus in male rats (109). In Rhesus monkeys, CSF OT levels were positively correlated with adult female age (110). Several authors did not find correlation between age and changes in the OT system and those reports mentioned comparable social memory and anti-depressant effect of OT injections and similar OT fiber density in the rat and a similar number of OT cells in the PVN in the human being (111–113). Considering a growing interest in elucidating neuroendocrine bases of behavioral modifications that occur during aging that are detrimental to the general health status, a thorough investigation of age-related neural changes – including those pertaining to the OT system – are of critical importance. We therefore stress the need to accelerate research on in-depth identification of age-related changes of central OT pathways as well as the functional link between central OT and alterations in macronutrient-driven reward in the aging process in order to aid in conceptualizing new diagnostic markers of pathophysiology of aging and in devising novel treatment strategies that integrate multiple functional facets of OT.

## Conflict of Interest Statement

The authors declare that the research was conducted in the absence of any commercial or financial relationships that could be construed as a potential conflict of interest.
